# Computational multifactoriality in a detailed neural network model resembling centre-surround suppression deficits in schizophrenia

**DOI:** 10.1186/1471-2202-15-S1-P1

**Published:** 2014-07-21

**Authors:** Christoph Metzner, Achim Schweikard, Bartosz Zurowski

**Affiliations:** 1 Institute for Robotics and Cognitive Systems, University of Luebeck, 23538 Luebeck, Germany; 2 Graduate School for Computing in Medicine and Life Sciences, University of Luebeck, 23538 Luebeck, Germany; 3 Department of Psychiatry, University of Luebeck, Schleswig-Holstein, 23538 Luebeck, Germany

## 

Over the last decades an enormous amount of studies have been conducted searching for biomarkers of psychiatric disorders on the molecular level, aiming at improving diagnostics and therapy thereof. However, the gap between the genetic/molecular scale and the behavioural scale is huge and genes with substantial contributions have not been found for most disorders. Furthermore, computational studies in neural networks have shown that different variations in cell and connection parameters can lead to distinguishable network activity [1,2]. To further explore the suggested multifactorial nature of psychiatric disorders, we present a study investigating centre-surround suppression (CSS)using a detailed multi-compartment network model of primary visual cortex V1 [4]. CSS refers to the mutual suppression of the neural representations of stimuli presented in the centre and in the surround of the visual or receptive field resulting perceptually in suppression of the centre stimulus. This effect has been shown impaired in schizophrenia [3]. CSS deficits as relying on inhibitory GABAergic neurotransmission has been recently incorporated in neurobiological models of schizophrenia focusing on GABAergic and glutamatergic dysfunction.

The present model is based on a previous model [4] that shows classical receptive field properties such as orientation/direction, spatial frequency and temporal frequency selectivity in good agreement with experimental data. Furthermore, this model shows CSS strength for visual contrast in response to sinusoidal gratings comparable with electrophysiological and psychophysical data [4]. Here we tested the CSS properties of the network model in response to centre-surround random contrast texture patterns as used in psychophysical procedures [3]. Next, we altered various parameters governing inhibition in the network in order to produce CSS properties in response to random texture patterns resembling observations from psychophysical studies in schizophrenic patients. In particular connection strength (both inhibitory-excitatory, and excitatory-inhibitory), number of connections (both inhibitory-excitatory, and excitatory-inhibitory), number of inhibitory neurons as well as the time constant of GABAergic synapses have been systematically varied. Together, a number of 20 variations was tested.

The introduction of a high contrast surrounding leads to a significant reduction of the neural response and produces CSS strengths comparable to psychophysical data in healthy subjects [3]. However, we find that a relatively high percentage of parameter variations (80%, 16/20) lead to CSS strengths in agreement with psychophysical findings in schizophrenic patients. Most of these parameter variations (81.25%, 13/16) moreover produce physiologically plausible firing rates and show classical receptive field properties.

We demonstrated multifactoriality in a model of perceptual deficits in schizophrenia focusing on CSS. Specifically we have shown that several different combinations of parameters produce equivalent CSS impairments. Our simulations suggest that there are various ways to implement a network that produces schizophrenia-like phenotypic properties on the perceptual level. For example, one might consequently expect that both, a reduced density of (parvalbumine expressing) GABAergic inhibitory interneurons as well as a dysfunction thereof may produce equivalent and (psychophysically) indistinguishable effects on CSS and, possibly, associated cognitive functions. Finally it should be noted that due to the complexity of the model only a coarse sampling of the large parameter space was presented here. A more fine-grained sampling is ongoing work.
